# Editorial: Oral neutrophils - the good, the bad, and the ugly

**DOI:** 10.3389/fimmu.2023.1225210

**Published:** 2023-05-24

**Authors:** Ljubomir Vitkov, Martin Herrmann, Jasmin Knopf

**Affiliations:** ^1^ Clinic of Operative Dentistry, Periodontology and Preventive Dentistry, Saarland University, Homburg, Germany; ^2^ Department of Environment & Biodiversity, University of Salzburg, Salzburg, Austria; ^3^ Department of Dental Pathology, University of East Sarajevo, East Sarajevo, Bosnia and Herzegovina; ^4^ Department of Internal Medicine 3 - Rheumatology and Immunology, Friedrich-Alexander-University Erlangen-Nürnberg (FAU) and Universitätsklinikum Erlangen, Erlangen, Germany; ^5^ Deutsches Zentrum für Immuntherapie (DZI), Friedrich-Alexander-University Erlangen-Nürnberg and Universitätsklinikum Erlangen, Erlangen, Germany; ^6^ Department of Pediatric Surgery, University Medical Centre Mannheim, University of Heidelberg, Mannheim, Germany

**Keywords:** dysbiosis, systemic low-grade inflammation, innate immunity, dysregulated immunity, maladaptive trained immunity

## 1 Introduction

Historically, all oral mucosa diseases due to non-specific symbiotic flora have been considered topical diseases. In the last two decades, this vision has been embodied by the idea of dysbiosis. However, the concept of dysbiosis as a pathogenic factor became very controversial in the last decade (1). Otherwise, the host microbiome is presently seen as the normal environment of the host, whereby both are in state of symbiosis and permanent interactions. The host controls its symbionts and if this fails, disease or even host death may occur. The control on symbionts is accomplished by both innate and adaptive immunity. In the oral mucosa the major defenders of the innate immune system are neutrophils and neutrophil extracellular traps (NETs). So, non-specific oral mucosa inflammation is caused by the symbiont flora when the innate immunity is dysregulated, due to either inborn immune defects, or acquired ones. In both cases, these immune defects can affect neutrophils. The former are characterised by insufficiency or lack of neutrophils in gingiva (2, 3) or by reduced NET formation on oral mucosal surfaces (4, 5), the latter by heavy neutrophil infiltration (6) and neutrophil hyper-responsiveness (7, 8). Over the last ten years, the conception of trained immunity (TI) has been developed (9, 10) and the inadequate host response has been denoted maladaptive TI (11). TI confers partial “autonomy” on neutrophils that does not underlie the direct control of adaptive immunity and dysregulated TI can harm gingiva and periodontium (12). The maladaptive TI, which is epigenetically encoded in haematopoietic stem and progenitor cells (HSPCs), causes neutrophil hyper-responsiveness. The latter dysregulates their immune response, which underlies the pathogenesis of periodontitis with late-onset. Transfusion of HSPCs, which are responsible for TI, from mice with ligature-induced periodontitis into healthy animals causes periodontitis, even without dysbiosis (8). Periodontitis with late-onset affects more than 60% of humans (13) and represent the largest share of all oral mucosal diseases. Periodontitis, its pathogenesis, the role of neutrophils, NETs and maladaptive TI has been reviewed by Vitkov et al. The neutrophil proneness to form NETs on mucosal surfaces as well as NET roles, detection, and visualisation have been described by Li et al.

## 2 Interconnection between oral disorders and other diseases

As periodontitis is a systemic low-grade inflammatory (LGI) disease (12, 14), implications can be expected in other organs. Irwandi et al. discussed the contribution of periodontitis to the onset, progression, and complications of atherosclerotic cardiovascular diseases. In addition, infectious diseases characterised by overproduction of NETs, like COVID-19 (15), are also aggravated in periodontitis (16), and is caused by maladaptive TI (17). Heterogeneity of neutrophils and inflammatory responses in patients with COVID-19 and healthy controls have been studied by Xu et al., Hornigold et al. reported the age-related decline in the resistance of mice to bacterial infection and in LPS/TLR4 pathway-dependent neutrophil responses. The implications of this senescence-related decline in responsiveness may contribute to exacerbating the transient bacteraemia associated with periodontitis (12). Tang et al. demonstrated that in acute stress the enriched transcripts were mainly related to inflammation, defence, wounding, wound healing, complement activation and pro-inflammatory cytokine production. Additionally, the concentration of IL-1b, IL-6 and neutrophil number in peripheral blood increased significantly after acute stress, indicating the immunity transition into an inflammatory state. In sum, acute stress led to rapid mobilisation of the immune system. The body presented an inflammatory state dominated by an innate immune response represented by neutrophils. These findings suggest that stress may be an important contributor to onset and progression of periodontitis. Siddiqui et al. studied the periodontal neuropeptide Substance P (SP) and its role on host responses and bone loss in ligature-induced periodontitis. Deletion of tachykinin precursor 1 (Tac1), a gene encodes SP, or treatment of gingiva with SP antagonist significantly reduced bone loss in ligature-induced periodontitis. Ligature-induced recruitment of leukocytes, including neutrophils, and increase in cytokines leading to bone loss in periodontium was significantly lower in Tac1 knockout mice. Furthermore, intragingival injection of SP, but not neurokinin A, induced a vigorous inflammatory response and osteoclast activation in alveolar bone and facilitated bone loss in ligature-induced periodontitis. These data suggest that the periodontal innervation, in particular SP, plays a significant role in regulating host responses and bone resorption in ligature-induced periodontitis.

## 3 Treatment strategies

With immunological advances, a number of treatment strategies have been introduced to combat neutrophil inflammatory responses and to reverse maladaptive TI. This enables geroscientific strategies for periodontal rejuvenation and periodontal bone restoration (18). Another treatment possibility in periodontitis might be suppression of NET formation. As summarised by Liu et al., therapies targeting NETs can be segmented into two categories: degradation/destabilization of NETs, and the inhibition of NETs formation. Due to adverse side effects, most treatment options are not justified for the treatment of periodontitis.

In patients with antithrombotic prophylaxis employing low molecular weight heparins, a possible benefit for periodontitis can be expected, as they reportedly reduce NETs formation and dissociate histones from the chromatin backbone of NETs, as resumed by Liu et al. However, any clinical studies on low molecular weight heparins in periodontitis are lacking. Irwandi et al. summarised that novel and targeted approaches to manipulate neutrophil numbers and functions are warranted within the context of the treatment of periodontitis and also to mitigate its potential impact on other LGI disease. However, manipulation of neutrophil numbers is no mild treatment option, as demonstrated by Hebeda et al. neutrophil depletion promotes a higher frequency of monocytes, natural killers, and T regulatory cells, and lower frequency of cytotoxic T cells in the blood.

Additional completely new treatment approaches have been reported. Li et al. studied inhibition effects of taurine on bacteria-induced NADPH oxidase-dependent neutrophil extracellular traps via TAK1/MAPK signalling pathways. Chen et al. analysed how the ginsenoside Rg5 counteracts NETosis and inflammatory response in neutrophils via the adenosine diphosphate receptor P2RY12 on the platelet surface, which may pave the road for its clinical application in inflammatory disorders. Cxcr2 plays a crucial role in phagocytic ability, reactive oxygen species production, F-actin and α-tubulin levels, and phosphorylation of ERK1/2 and p38 MAPK, impaired PI3K-AKT, NF-κB, TGFβ and IFNγ pathways. Therefore, Cxcr2 blockers might also be a possible option to attenuate the neutrophil hyper-responsiveness seen in periodontitis Delobel et al.

## 4 Conclusion

Recently, the attention in mucosal inflammatory disease has been shifted from the topical to systemic point of view i.e., from the concept of dental biofilm dysbiosis to that of dysregulated immunity. The oral dysbiosis appears to be rather a corollary phenomenon of dysregulated immunity. The neutrophils and NETs play a crucial role in the mucosal inflammatory diseases. Reversing the maladaptive TI and geroscientific strategies may aid in oral mucosal rejuvenation and even reversion of periodontal bone loses, as recently demonstrated in experimental animals. The prophylaxis and treatment perspectives of oral mucosal pathology particularly periodontology largely depends on the progress in neutrophil immunology.

## Author contributions

All authors listed have made a substantial, direct and intellectual contribution to the work, and approved it for publication.
